# Astrocyte-derived phosphatidic acid promotes dendritic branching

**DOI:** 10.1038/srep21096

**Published:** 2016-02-17

**Authors:** Yan-Bing Zhu, Weizhen Gao, Yongbo Zhang, Feng Jia, Hai-Long Zhang, Ying-Zi Liu, Xue-Fang Sun, Yuhua Yin, Dong-Min Yin

**Affiliations:** 1 Laboratories of Stem Cell Biology and Regenerative Medicine, Department of Neurology, Experimental Research Center, Beijing Friendship Hospital, Capital Medical University, Beijing, China; 2 Department of Neurosurgery, Renji Hospital, Shanghai Jiao Tong University School of Medicine, Shanghai, China; 3 Key Laboratory of Brain Functional Genomics, Ministry of Education, Shanghai Key Laboratory of Brain Functional Genomics, School of Life Sciences, East China Normal University, Shanghai, China

## Abstract

Astrocytes play critical roles in neural circuit formation and function. Recent studies have revealed several secreted and contact-mediated signals from astrocytes which are essential for neurite outgrowth and synapse formation. However, the mechanisms underlying the regulation of dendritic branching by astrocytes remain elusive. Phospholipase D1 (PLD1), which catalyzes the hydrolysis of phosphatidylcholine (PC) to generate phosphatidic acid (PA) and choline, has been implicated in the regulation of neurite outgrowth. Here we showed that knockdown of PLD1 selectively in astrocytes reduced dendritic branching of neurons in neuron-glia mixed culture. Further studies from sandwich-like cocultures and astrocyte conditioned medium suggested that astrocyte PLD1 regulated dendritic branching through secreted signals. We later demonstrated that PA was the key mediator for astrocyte PLD1 to regulate dendritic branching. Moreover, PA itself was sufficient to promote dendritic branching of neurons. Lastly, we showed that PA could activate protein kinase A (PKA) in neurons and promote dendritic branching through PKA signaling. Taken together, our results demonstrate that astrocyte PLD1 and its lipid product PA are essential regulators of dendritic branching in neurons. These results may provide new insight into mechanisms underlying how astrocytes regulate dendrite growth of neurons.

Astrocytes have recently emerged as key regulators of brain circuit formation and function. Recent studies have demonstrated that astrocytes regulate synapse formation through secreted and contact-mediated signals^1^. Besides synapse formation, dendrite morphogenesis is another important step for neural circuit development. The numbers of primary dendrites arising from the cell body, higher order dendrites emerging from primary dendrites, and dendritic branching patterns appear to be critical for neuronal function^2^. It has long been recognized that astrocytes could promote neurite outgrowth^3^,^4^. Several studies have identified various diffusible and non-diffusible proteins from astrocytes to mediate neurite outgrowth^5^,^6^,^7^,^8^. However, relatively little is known about the lipid molecules from astrocytes essential for the regulation of dendritic branching.

Phospholipase D (PLD), which catalyzes the hydrolysis of phosphatidylcholine (PC) to generate phosphatidic acid (PA) and choline, has been implicated in the regulation of neurite outgrowth^9^,^10^,^11^. PLD isozymes, including PLD1 and PLD2, are expressed in both neuron and glia cells in the brain^12^. PLD null mutant mice showed impaired brain development and reduced cognitive function^13^. Our previous studies demonstrated that knockdown of PLD1 from individual neurons increased dendritic branching through cell autonomous mechanisms^14^. By contrast, a recent study found that dendritic branching was reduced in PLD1 null mutant mice^15^ where PLD1 is deleted from both neurons and astrocytes. Since PLD1 is highly expressed in astrocytes^16^, these apparently contradictory observations lead us to investigate whether PLD1 from astrocytes plays any roles in dendritic branching of neurons.

In the present study, we used mixed culture composed of neuron and glia to study the roles of astrocyte PLD1 in dendritic branching. We found that knockdown of PLD1 only in astrocytes reduced dendritic branching of neurons in mixed culture. Further study from sandwich-like coculture and astrocyte conditioned medium suggested that astrocyte PLD1 regulated dendritic branching through secreted signals, which was evidenced by the observation that PA could rescue the dendritic deficits of neurons in mixed and sandwich-like coculture where PLD1 was selectively reduced in astrocytes. Moreover, PA itself is sufficient to promote dendritic branching of neurons. Finally we showed that PA increased dendritic branching by activation of protein kinase A signaling in neurons. Taken together, these results demonstrate that PLD1-mediated secretion of PA from astrocytes is essential for dendritic branching in neurons.

## Results

### The protein levels of PLD1 were higher in astrocytes than in neurons

**Figure 1:**
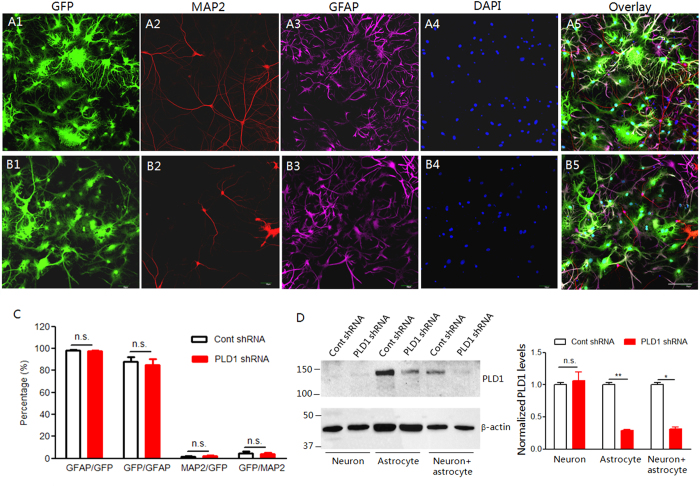
Selective knockdown of PLD1 in astrocytes. (**A–B**) Immuostaining of GFP, MAP2, GFAP, and DAPI in DIV 15 neuron-glia mixed culture infected by lentivirus expressing control (A1-5) and PLD1 shRNA (B1-5). Bar, 100 μm. (**C**) quantification of the percentage of astrocyte or neuron in GFP-positive cells and the percentage of astrocyte or neuron infected by lentivirus. (**D**) Western blot analysis of PLD1 from neuronal, astrocyte and neuron-astrocyte mixed culture infected by lentivirus expressing control or PLD1 shRNA, left, representative images, right, quantification data, the protein level of PLD1 was normalized by that in control shRNA group, *p < 0.05, **p < 0.01, n = 4 samples per group.

**Figure 2:**
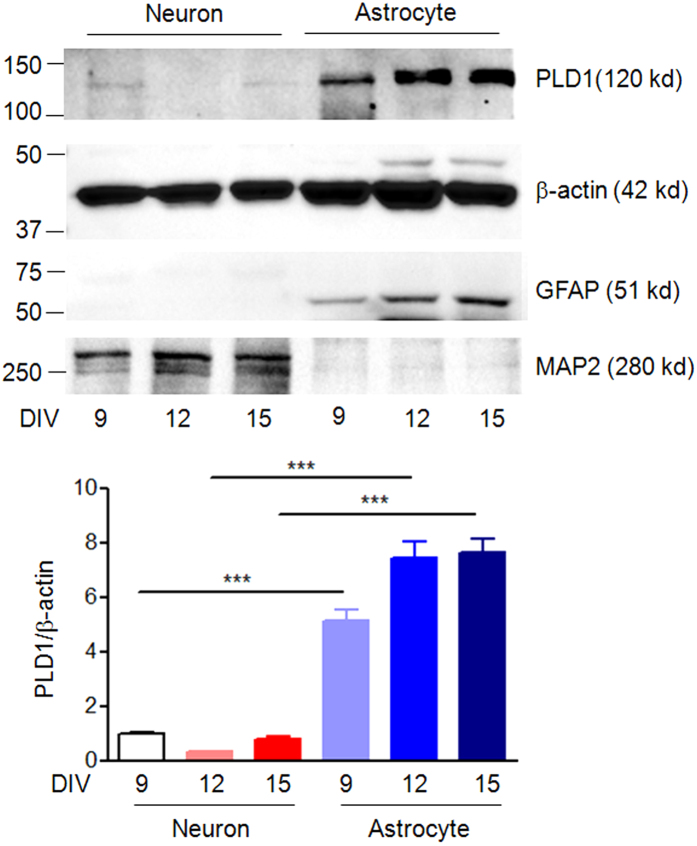
Western blot analysis of PLD1, β-actin, GFAP and MAP2 from primary cultures of neuron and astrocyte at indicated time points. Top, representative images, bottom,quantification data, n = 3 per sample, ***p < 0.001, between neuron and astrocyte.

To study the roles of astrocyte PLD1 in dendritic branching of neurons, we took use of the neuron-glia mixed culture from embryonic day 18 (E18) rat hippocampus. As shown in Figure S1, the astrocyte density is very low at days *in vitro* (DIV) 3 in our hippocampal neuron-glia mixed culture and thus the effects of astrocytes on dendritic branching before DIV 3 may be very weak. In contrast, the astrocyte density reached a relatively high level after DIV9 in the mixed culture and the ratio of astrocytes to neurons reached 8:1 at DIV 15 (Fig. S1, Fig. 1 (A1-5 and B1-5)), which is close to the *in vivo* conditions (as stated 10:1 in Eric Kandel *et al*., the Principles of Neural Science). Due to these reasons, our study focused on the time window between DIV 9 and 15. We first compared PLD1 protein levels from neuronal and astrocyte culture at DIV 9, 12 and 15. Strikingly, the protein levels of PLD1 in astrocytes are in average 7-fold higher than that in neurons between DIV 9 and 15 (Fig. 2), which provided a clue for the importance of PLD1 from astrocytes.

To study the roles of astrocyte PLD1 in dendritic branching, we generated lentivirus expressing PLD1 short hairpin RNA (shRNA) or non-silencing (control) shRNA using the shuttle vector pLKD.CMV.GFP.U6.shRNA which has a CMV promoter. The lentivirus we generated did not infect neurons in the neuron-glia mixed culture as indicated by the non-overlap between GFP and MAP2, a protein marker for neurons (Fig. 1A1-5). In contrast, most of the cells infected by lentivirus were astrocytes as evidenced by the co-localization of GFP and GFAP, a protein marker for astrocytes (Fig. 1A1-5). The quantification assay showed that 98% of cells infected by lentivirus were astrocytes and only 2% of GFP-positive cells were neurons (Fig. 1C). On the other hand, nearly 90% of astrocytes were transduced by lentivirus and expressed GFP (Fig. 1C). Moreover, the lentivirus expressing PLD1 shRNA did not alter the neuronal PLD1 expression which was pretty low under control conditions (Fig. 1D). In contrast, the lentivirus expressing PLD1 shRNA significantly reduced PLD1 expression in astrocyte and neuron-astrocyte mixed cultures (Fig. 1D). All these results demonstrated both the specificity and efficiency of the lentivirus in transduction of astrocytes.

### Knockdown of PLD1 in astrocytes reduced PA secretion and dendritic branching

**Figure 3:**
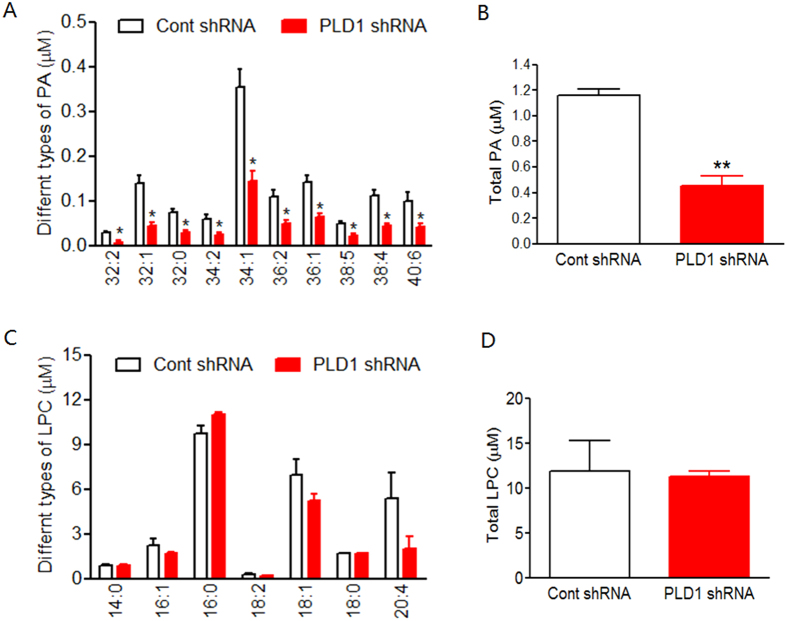
Concentration of PA and LPC in DIV 15 astrocyte cultured medium (ACM) infected by lentivirus expressing control and PLD1 shRNA. (**A**) concentration of different types of PA, *p < 0.05, n = 3 different experiments. (**B**) concentration of total PA listed in (panel **A**), *p < 0.05, n = 3 different experiments. (**C**) concentration of different types of LPC. (**D**) concentration of total LPC listed in panel **C**.

PLD has been shown to be critical for the exocytosis of PA-containing vesicles^17^,^18^,^19^ and thus PA may be released together with the secretory vesicles. We first studied whether downregulation of PLD1 by shRNA affected the secretion of PA from astrocytes. For this purpose, cultured astrocytes were transduced by the lentivirus expressing PLD1 or control shRNA at DIV 9 and the astrocyte culture medium (ACM) were collected and analyzed at DIV 15. As shown in Fig. 3A,B, the concentration of PA in control ACM reached 1.16 μM and PLD1 shRNA significantly reduced PA levels to 0.45 μM. By contrast, the levels of another phospholipid lyso-phosphatidylcholine (LPC) in ACM were not altered by PLD1 shRNA (Fig. 3C,D) demonstrating the specificity of PLD1 shRNA.

**Figure 4:**
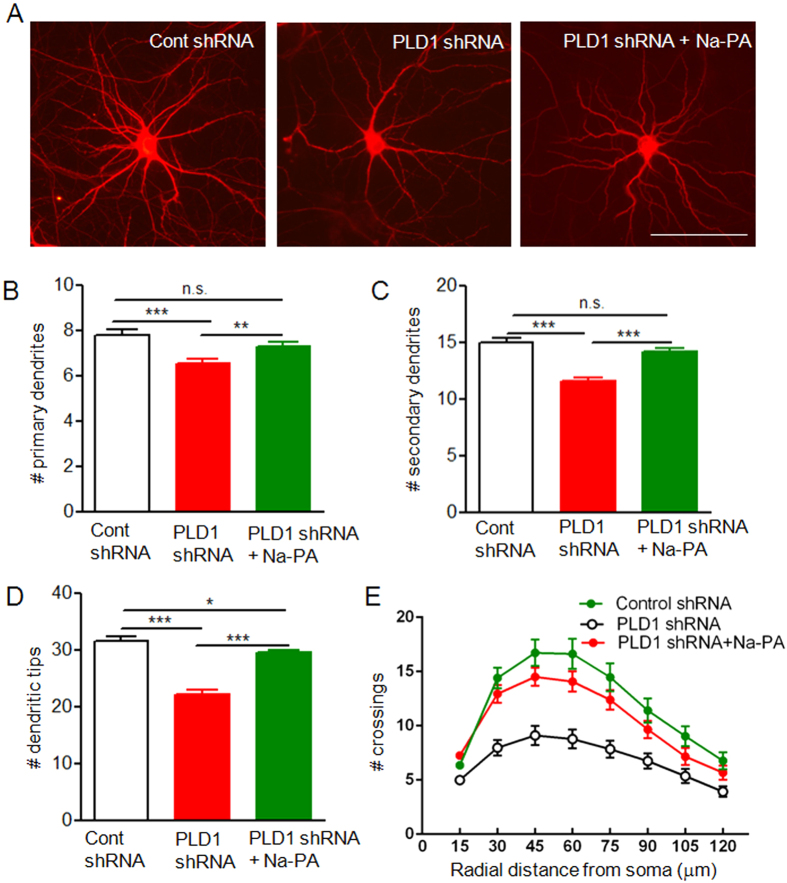
Dendritic branching of neurons in neuron-glia mixed culture. (**A**) MAP2 staining of DIV 15 neurons in neuron-glia culture where only astrocytes were infected by lentivirus expressing control shRNA, PLD1 shRNA with and without 1 μM Na-PA in the culturing medium. Bar, 100 μm. (**B–E**) Quantification of primary dendrites (**B**), secondary dendrites (**C**), dendritic tips (**D**) and Sholl analysis (**E**) for three groups of neurons in panel **A**, n = 153 neurons for control shRNA, n = 186 neurons for PLD1 shRNA, n = 216 neurons for PLD1 shRNA plus Na-PA from 3 independent experiments, (**B**–**D**) *p < 0.05, **p < 0.01, ***p < 0.001, E, F (2, 1536) = 354.2, p < 0.001.

Next we investigated the role of astrocyte PLD1 in dendritic branching. The neuron-glia mixed culture was infected by the lentivirus expressing PLD1 or control shRNA at DIV 9 and the cells were fixed and analyzed at DIV 15. To differentiate dendrites and axon, we stained the neurons with anti-MAP2, a protein marker for dendrites. Only MAP2-positive neurites were selected for further analysis of dendritic branching. The number of astrocytes and neurons were comparable between control and PLD1 shRNA treated cultures (Fig. S2). However, the dendritic branches were reduced in neuron-glia mixed culture when astrocyte PLD1 was downregulated as reflected by lower number of primary and secondary dendrites (Fig. 4A–C). Because the dendritic tips were at the terminal branches and reflected the complexity of dendrites, we next analyzed whether the number of dendritic tips were decreased after downregulation of PLD1 in astrocytes. Consistent with the results from primary and secondary dendrites, the number of dendritic tips was reduced in neuron-glia mixed culture after knockdown of PLD1 expression in astrocytes (Fig. 4D). Finally, the essential role of astrocyte PLD1 in supporting dendritic branching was confirmed by Sholl analysis (Fig. 4E), which is the standard assay of dendritic complexity^20^. All these results suggest that PLD1 in astrocytes is essential for dendritic branching of neurons. Transfection with a fluorescence DNA is a convenient way to identify individual neurons as we did before^14^,^21^. To differentiate with GFP expressed by the lentivirus, the neuron-glia mixed culture was transfected with dsRed plasmids. The results from dsRed transfection showed that selective knockdown of PLD1 from astrocytes reduced dendritic branching in neuron-glia mixed culture with both low and normal densities (Figures S3 and S4). Since the overall conclusion from dsRed transfection (Figures S3 and S4) is consistent with that from MAP2 staining (Fig. 4), we used MAP2 staining in low density cultures for the later experiments.

PLD1 hydrolyses PC to produce choline and PA. While the newly synthesized choline is a small, organic and water-soluble molecule, most of the physiological functions of PLD1 are exerted by PA^22^. To demonstrate whether the effects of astrocyte PLD1 on dendritic branching is through PA, we added sodium phosphatidic acid (Na-PA), a sodium salt of PA into the medium of neuron-glia mixed culture at DIV12, 3 days after the application of lentivirus expressing PLD1 shRNA. Na-PA was continuously present in the culturing medium and thus overcame the reduction of PA released from PLD1 knockdown astrocytes. Na-PA increased dendritic branching of neurons cocultured with PLD1 knockdown astrocytes to the very close level as that cocultured with control astrocytes (Figures 4 and S3). PA can be hydrolyzed to lyso-PA (LPA), a lipid mediator that has 5 well described receptors^23^. But the amount of LPA in ACM is too low to be detected and LPA did not rescue the dendritic phenotype after PLD1 downregulation in astrocytes (Fig. S3). These results indicate that astrocyte PLD1 regulates dendritic branching largely through PA.

### Astrocyte PLD1 regulated dendritic branching in sandwich-like coculture

**Figure 5:**
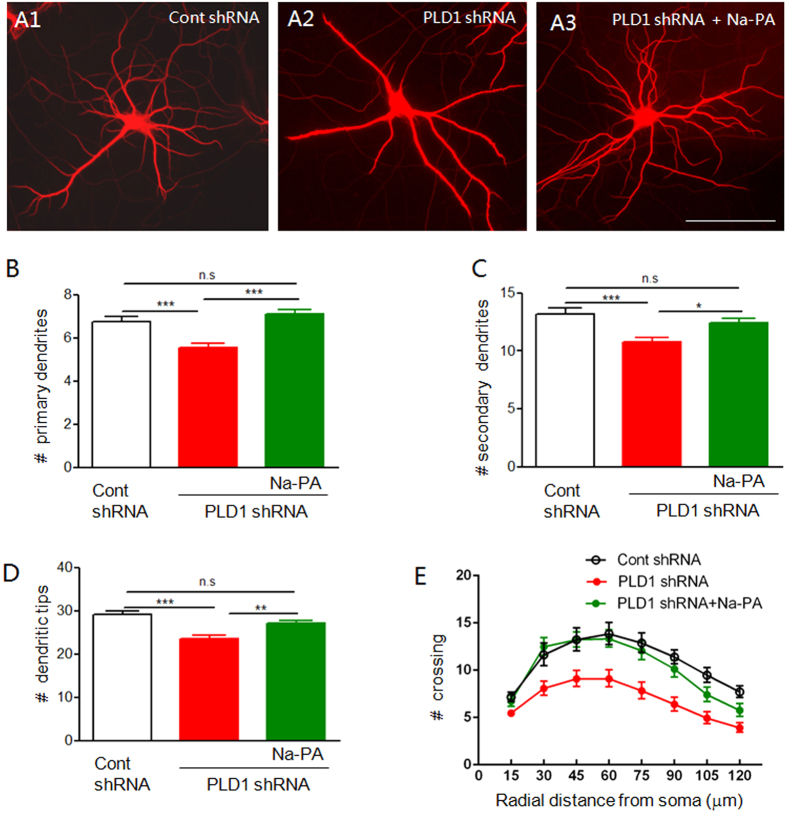
Dendritic branching of neurons grown in sandwich-like coculture. (**A1**–**3**) MAP2 staining of DIV 15 neurons in sandwich-like coculture where astrocytes were infected by lentivirus expressing control shRNA, PLD1 shRNA with and without 1 μM Na-PA treatment. Bar, 100 μm. (**B**–**E**) Quantification of primary dendrites (**B**), secondary dendrites (**C**), dendritic tips (**D**) and Sholl analysis (**E**) for three groups of neurons in panel **A**, n = 55 neurons for control shRNA, n = 62 neurons for PLD1 shRNA, n = 71 neurons for PLD1 shRNA plus Na-PA, (**B**–**D**) *p < 0.05, **p < 0.01, ***p < 0.001, E, F (2, 1224) = 238.5, p < 0.001.

It is possible that astrocytes promote dendritic branching of neurons through secreted or contact-mediated signals. Since PA can rescue the dendritic deficits caused by PLD1 downregulation in astrocytes, the regulation of dendritic branching by astrocyte PLD1 may be via secreted signals. To test this hypothesis, we took use of the sandwich-like coculture^24^ in which astrocytes and neurons are not physically interacted. Strikingly, knockdown of PLD1 in astrocytes significantly reduced the dendritic branching of neurons in sandwich-like coculture (Fig. 5). These data suggest that the regulation of dendritic branching by astrocyte PLD1 does not require the direct contact between astrocytes and neurons and might be through secreted signals from astrocytes. In support of this notion was the observation that feeding the coculture where astrocyte PLD1 was downregulated with Na-PA could rescue the deficits in dendritic branching of neurons (Fig. 5).

### Neurons grown in conditioned medium from PLD1 knockdown astrocytes had less dendritic branching

**Figure 6:**
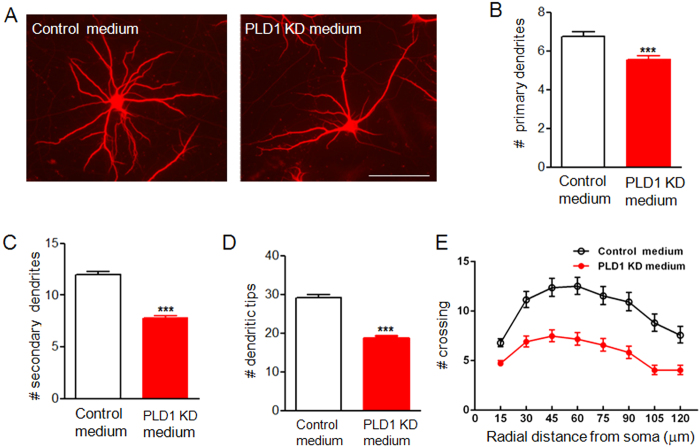
Dendritic branching of neurons grown in astrocyte conditioned medium. (**A**) MAP2 staining of DIV 15 neurons grown in conditioned medium from control or PLD1 knockdown astrocytes. Bar, 100 μm. (**B–E**) Quantification of primary dendrites (**B**), secondary dendrites (**C**), dendritic tips (**D**) and Sholl analysis (**E**) for two groups of neurons in panel A, n = 60 neurons in control medium, n = 88 neurons in PLD1 knockdown (KD) medium, (**B**–**D**) ***p < 0.001, E, F (1, 1096) = 652.8, p < 0.001.

The above data suggested that astrocyte PLD1 regulated dendritic branching through secreted signals. To confirm that the effects of astrocyte PLD1 on dendritic branching do not require any co existence between neuron and glia, we applied the conditioned medium from control and PLD1 knockdown astrocytes into pure neuronal culture. Control and PLD1 knockdown astrocytes were infected by lentivirus expressing control and PLD1 shRNA at DIV 9. The astrocyte conditioned medium was collected at DIV 15 to be consistent with the studies from neuron-glia mixed culture. Neurons grown in conditioned medium from control astrocytes have more dendritic branching than that grown in conditioned medium from PLD1 knockdown astrocytes (Fig. 6). These results confirmed our previous finding that astrocyte PLD1 promoted dendritic branching through secreted signals but not dependent on the physical interaction between neuron and glia.

### Inhibition of PLD1 reduced dendritic branching of neurons in neuron-glia mixed culture

**Figure 7:**
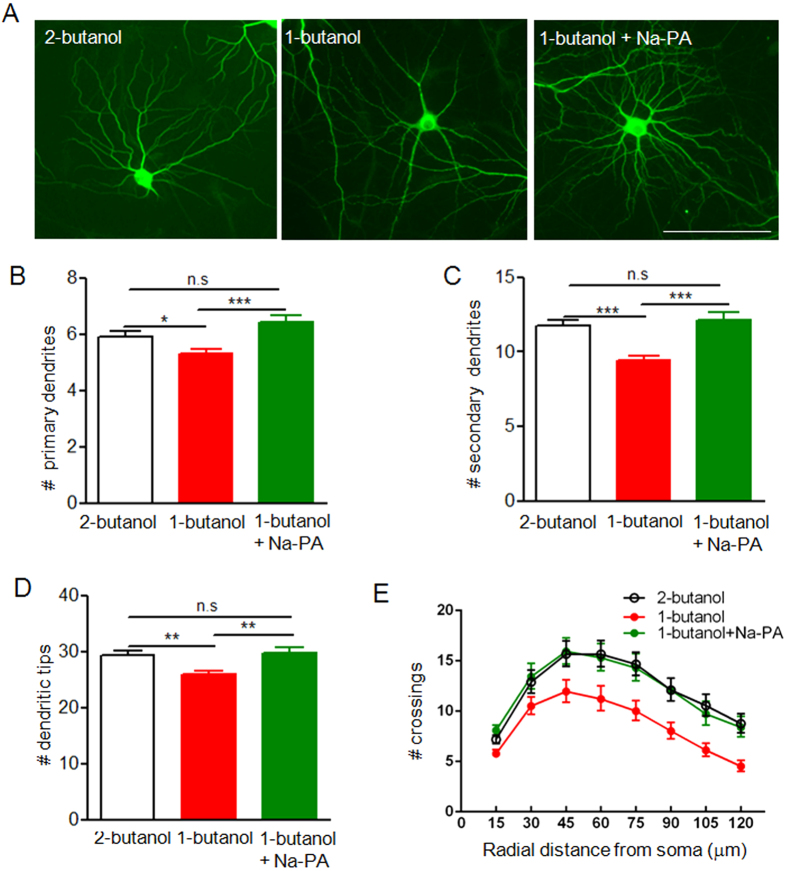
Dendritic branching of neurons in neuron-glia mixed culture under treatment with vehicle, PLD1 inhibitor and Na-PA. (**A**) MAP2 staining of DIV 15 neurons in neuron-glia mixed culture treated with 2-butanol, 1-butanol or 1-butanol plus 1 μM Na-PA. Mixed culture was treated with 0.5% 2-butanol, 0.5% 1-butanol or 0.5% 1-butanol plus 1 μM Na-PA at DIV 9. After 6 days of treatment the cells were fixed and subjected to immunostaining with anti-MAP2 antibodies. Bar, 100 μm. (**B–E**) Quantification of primary dendrites (**B**), secondary dendrites (**C**), dendritic tips (**D**) and Sholl analysis (**E**) for three groups of neurons in panel A, n = 54 for neurons treated with 2-butanol , n = 63 for neurons treated with 1-butanol, n = 44 for neurons treated with 1-butanol plus Na-PA. (**B**–**D**) *p < 0.05, **p < 0.01, ***p < 0.001, E, F (2, 1064) = 142.4, p < 0.001.

Next we determine whether inhibition of PLD1 could have the same effects as PLD1 knockdown on dendritic branching. To this end, we treated neuron-glia mixed culture with 1-butanol, an inhibitor of PA synthesis^25^,^26^ at DIV 9 and analyzed the dendritic branching at DIV 15. We found that neurons in mixed culture treated with 1-butanol have less dendritic branches than that under the treatment with 2-butanol, the inactive analog of 1-butanol (Fig. 7). Strikingly, Na-PA fully rescued the reduced dendritic branching of neurons in mixed culture treated with 1-butanol (Fig. 7). By contrast, 1-butanol has no detrimental effect on dendritic arborisation in pure neuronal culture where neurons are the unique source of PA (Fig. S5). Together these results demonstrated that PA from astrocytes is important for the late stage of dendritic arborisation between DIV 9 and 15.

### PA promoted dendritic branching in pure neuronal culture

**Figure 8:**
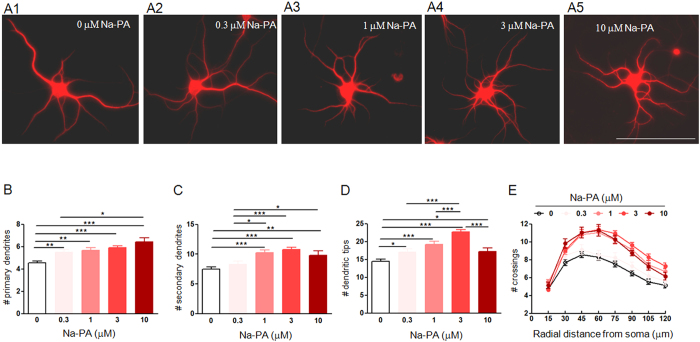
Dendritic branching of neurons in pure neuronal culture under treatment with vehicle or different dosages of Na-PA. (**A–E**) MAP2 staining of DIV 12 neurons in pure neuronal culture treated with vehicle (A1), 0.3 μM (A2), 1 μM (A3), 3 μM (A4) or 10 μM Na-PA (A5). Bar, 100 μm. (**B–E**) Quantification of primary dendrites (**B**), secondary dendrites (**C**), dendritic tips (**D**) and Sholl analysis (**E**) for five groups of neurons in panel A1 to 5, n = 84 for neurons treated with vehicle; n = 73, 78, 82, 68 for neurons treated with 0.3, 1, 3 and 10 μM Na-PA, B-D, *p < 0.05, **p < 0.01, ***p < 0.001, E, F (4, 1552) = 62.54, p < 0.001.

All the above-mentioned results suggest that PLD1 mediated secretion of PA from astrocytes is essential for dendritic branching in neurons. Next we investigated whether PA itself was sufficient to promote dendritic branching in pure neuronal culture. Toward this end, we treated cultured neurons with vehicle and different dosages of Na-PA at DIV 9. Neurons were fixed and dendritic branching was analyzed at DIV 12. As shown in Fig. 8, 0.3 μM Na-PA slightly increased dendritic branching and 1 μM Na-PA exerted a medium effect between 0.3 μM and 3 μM Na-PA. However, 10 μM Na-PA did not further increased but reduced dendritic branching compared with 3 μM Na-PA. This kind of dose dependence underlying PA regulation of dendritic branching might reflect the binding property between PA and its receptors. Alternatively, chronic treatment with 10 μM Na-PA may have some toxic effects on neurons. Actually, we found that the neuron number under the treatment with 10 μM Na-PA was somewhat reduced compared with control and other group of neurons receiving lower dosage of Na-PA (data not shown).

### PA promoted dendritic branching via PKA signaling in neurons

**Figure 9:**
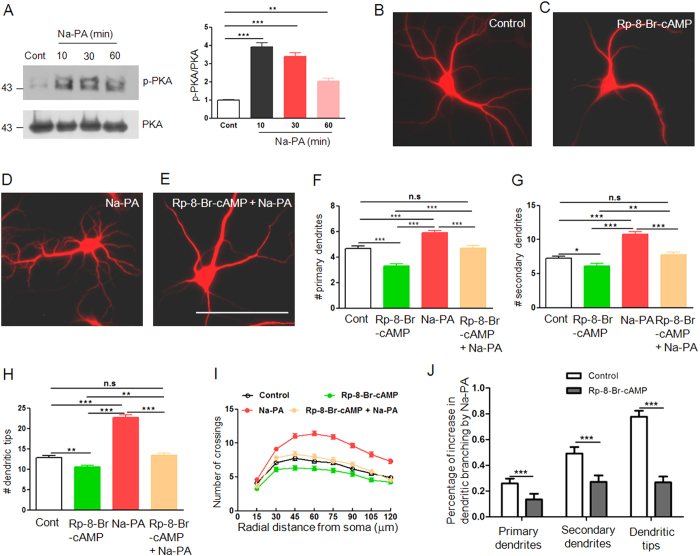
PKA activation and dendritic branching of neurons in pure neuronal culture under the treatment with Na-PA or PKA inhibitor or both. (**A**) Western blot analysis of Thr 198 phosphorylated PKA catalytic subunit (p-PKA) and total PKA catalytic subunit from DIV 12 neuronal lysate at different time points after Na-PA treatment, left, representative images, right, quantification data, the ratio of p-PKA to PKA was normalized by that in control neurons, ***p < 0.001, n = 3 independent experiments. (**B–E**) MAP2 staining of DIV 12 neurons in pure neuronal culture treated with vehicle (**B**), 40 μM Rp-8-Br-cAMP (**C**), 3 μM Na-PA (**D**) or 3 μM Na-PA plus 40 μM Rp-8-Br-cAMP (**E**). Bar, 100 μm. (**F–I**) Quantification of primary dendrites (**F**), secondary dendrites (**G**), dendritic tips (**H**) and Sholl analysis (**I**) for four groups of neurons in panel **B**–**E**, n = 88, 90, 95, 98, for neurons treated with vehicle, Rp-8-Br-cAMP, Na-PA or Na-PA plus Rp-8-Br-cAMP. F-H, *p < 0.05, **p < 0.01, ***p < 0.001, I, F (3, 1328) = 223.76, p < 0.001. (**J**) Quantification of the PA increase of dendritic branching in control and Rp-8-Br-cAMP treated neurons, ***p < 0.001.

Extracellular phospholipids usually exert their functions through G protein coupled receptors (GPCRs)^27^. Cyclic AMP (cAMP) and protein kinase A (PKA) are coupled with GPCRs and involved in neurite outgrowth^28^. Thus we first tested whether neuronal PKA can be activated by PA. To this end, we treated the pure neuronal culture with 3 μM Na-PA which showed maximal effects on dendritic branching (Fig. 8). An antibody against the PKA catalytic subunit phosphorylated at Thr 198 was used to detect the active form of PKA^29^. As shown in Fig. 9A, activity of neuronal PKA was increased upon Na-PA treatment for 10 minutes and dropped from the peak afterwards while the total level of PKA was not upregulated. Next we determined whether the activation of PKA is required for PA to promote dendritic branching. Toward this end, we applied Rp-8-Br-cAMP, a stable and membrane permanent PKA inhibitor^30^ into neuronal culture together with Na-PA at DIV 9 for 3 days. In agreement with previous finding, Na-PA promoted dendritic branching (Fig. 9B,D,F–I). Strikingly, the neurons treated with Na-PA and Rp-8-Br-cAMP have the same dendritic complexity as control neurons (Fig. 9B,E–I). Of note, Rp-8-Br-cAMP itself reduced dendritic branching (Fig. 9B,C,F–I) reflecting the necessity of endogenous PKA activity. Na-PA could still increase dendritic branching in the presence of Rp-8-Br-cAMP (Fig. 9C,E–I). However, Na-PA increased dendritic branching to a lesser extent in Rp-8-Br-cAMP treated neurons compared with control neurons (Fig. 9J). These results suggested that PKA activation was one of the major mechanisms underlying PA promotion of dendritic branching although other signaling pathways may also contribute.

## Discussion

Here we used three different culture systems to demonstrate that PLD1 from astrocytes is essential for dendritic branching of neurons. First we showed that knockdown of PLD1 selectively in astrocytes reduced dendritic branching of neurons in neuron-glia mixed culture, which is consistent with a recent report that dendritic branching was decreased in PLD1 null mutant mice^15^. The lentivirus expressing PLD1 shRNA has a CMV promoter and hence infected mostly astrocytes but not neurons. Thus, the effects of PLD1 knockdown on dendritic branching of neurons in neuron-glia mixed culture are apparently mediated by astrocytes. This notion was further supported by the results from sandwich-like coculture where astrocytes and neurons are separated. In this case PLD1shRNA was only expressed in astrocytes and led to the reduction in dendritic branching of neurons. Moreover, the neurons grown in conditioned medium from PLD1 knockdown astrocytes had less dendritic branching compared with neurons grown in normal astrocyte conditioned medium. These results further demonstrated that astrocyte PLD1 regulated dendritic branching of neurons through secreted signals. In support of PLD1 and PA role in astrocyte-neuron communication, it has previously been reported that these molecules take part in the transduction pathways that link muscarinic receptor activation in astrocytes to neuritogenesis in neighboring hippocampal neurons by acting upstream of PKC and NF-Kβ^31^.

PLD1 and PA have been implicated in astrocyte proliferation and PLD1 deficient astrocytes display reduced proliferation^13^,^32^. Burkhardt *et al*. performed primary culture of astrocytes from PLD1 null mutant mice and thus PLD1 proteins were deleted from astrocytes starting from DIV 0. In contrast, we infected astrocytes with lentivirus expressing PLD1 shRNA starting from DIV 9. Since shRNA from lentiviral vector usually takes 1–3 days to knockdown protein levels, the peak stage of astrocyte proliferation (from DIV 3 to 9, Fig. S1) has already been bypassed when PLD1 was downregulated in our conditions. For these reasons, transduction of astrocytes with lentivirus expressing PLD1 shRNA at DIV 9 did not significantly alter the astrocyte number at DIV 15 (Fig. S2).

PLD1 produces PA^33^ and has been implicated in neurite growth and neurotransmitter release^9^,^10^,^11^,^34^,^35^. Here we showed that PA could rescue the deficit in dendritic branching after knockdown of PLD1 in astrocytes, which demonstrated the necessity of PA. In addition, the dendritic branching of neurons was reduced when PA synthesis was inhibited by 1-butanol in neuron-glia mixed culture. Moreover, 1-butanol inhibition of dendritic branching in neuron-glia mixed culture was abolished by exogenous application of Na-PA. However, 1-butanol has no detrimental effect on dendritic arborisation in pure neuronal culture where neurons are the unique source of PA (Fig. S5). Lastly, Na-PA itself could promote dendritic branching in pure neuronal culture via a dose dependent manner. All these results suggest PA from astrocyte is important for the late stage of dendritic branching between DIV 9 and 15.

Our previous data showed that 1-butanol treatment in pure neuronal culture at DIV6 increased dendritic branching at DIV 9, reflecting a negative role for neuronal PLD1 in dendritic branching^14^. Of note, we have shown that PLD1 protein levels became decreased during the maturation of dendrites in pure neuronal culture^14^. Consistent with this notion is the observation that PLD1 protein levels in neurons are very lower after DIV 9 and hard to be detected at DIV 15 (Figures 1D and 2). In contrast, the protein levels of PLD1 in astrocytes are much higher than that in neurons after DIV 9 (Figures 1D and 2). The extremely low levels of PLD1 in neurons may explain the minor effects of neuronal PLD1 on dendritic branching at late stage between DIV 9 and 15 (Fig. S5).

Exactly how astrocyte-derived PA promotes dendritic branching and the neuronal receptors for PA are unknown. Extracellular phospholipids usually exert their functions through G protein coupled receptors (GPCRs)^23^,^27^ which are linked to PKA signaling^28^. We showed here that PKA was involved in PA increasing of dendritic branching. Of note, inhibition of PKA did not completely abolish the effects of PA on dendritic branching, which suggested that other signaling proteins besides PKA also contributed to PA promotion of dendritic branching. PA may tether a protein into cell membrane or modulate the catalytic activity of an enzyme through the direct binding^36^. It might be possible that astrocyte-derived PA bind with and stabilize the membrane proteins on neuronal surface to promote dendritic branching. Several membrane proteins such as cadherins and intergrins may be involved in astrocyte-regulated dendritic branching^6^,^37^. It remains interesting to study whether PA affects the membrane localization of cell adhesion molecules and/or regulates activity of enzymes that are critical for dendritic branching.

## Methods

### Antibodies and chemicals

We used the following antibodies and chemicals: rabbit polyclonal anti-microtubule associated protein 2 (MAP2) (AB5622; Millipore), mouse monoclonal anti-GFAP antibody (MAB360; Millipore), rabbit polyclonal anti-PLD1 (#3832; Cell Signaling Technology), mouse monoclonal anti-β-actin (A2228; Sigma-Aldrich), rabbit polyclonal anti-p-PKA catalytic subunit (Thr 198) (SC-32968; Santa Cruz), rabbit polyclonal anti-PKA catalytic subunit (SC-903; Santa Cruz), poly-D-lysine (Sigma-Aldrich), B27 and GlutaMAX-I (Invitrogen), cytosine arabinoside (Ara-C, Sigma-Aldrich), sodium phosphatidic acid (Na-PA, 840875P, Avanti Polar Lipids, Alabaster, AL), Rp-8-Br-cAMP (SC-3539A, Santa Cruz) and 1-butanol and 2-butanol were purchased from Sigma-Aldrich.

### Neuronal culture

Hippocampal explants isolated from embryonic day 18 (E18) rats were digested with 0.25% trypsin for 30 min at 37 °C followed by trituration with a pipette in plating medium (DMEM with 10% fetal bovine serum). Dissociated neurons were plated onto coverslips in 35 mm dishes coated with poly-D-lysine at a low density of 0.2 × 10^5^ per dish and a normal density of 2 × 10^5^ per dish. After culturing for 4 h, media were changed to Neurobasal medium supplemented with 2% B27 and 0.5 mM GlutaMAX-I. Neurons were grown in the medium with or without 5 μM Ara-C for pure neuronal culture and neuron-glia mixed culture, respectively. The sandwich-like coculture was performed as previously described^24^. Briefly, we first prepared astrocyte cultures from cerebral cortex of newborn rats. The astrocyte culture was infected by lentivirus at DIV 14 for 6 days. Then the DMEM medium containing lentivirus was replaced by neurobasal medium plus 2% B27 and 2 days later we transferred coverslips with DIV 9 neurons to the dishes with glial feeder. After 6 days of coculture, the neurons were fixed and immunostained with anti-MAP2.

### Astrocyte culture

Astrocyte cultures were prepared from the cerebral cortex of newborn rats as previously described^38^. Dissociated brain cells were seeded in 35 mm dishes and maintained in 2 mL DMEM with 10% fetal bovine serum. Cultures were fed twice a week. To generate astrocytic conditioned medium (ACM), the DMEM medium was replaced by 1 mL neurobasal medium at DIV 9 when the astrocytes were transduced by lentivirus expressing control or PLD1 shRNA. After another 6 days of culture the neurobasal medium was collected as ACM and added to the pure neuronal culture supplemented with 0.5% B27. The pure neuronal culture was treated with astrocyte conditioned medium at DIV 9 for 6 days. Then the cells were fixed and immunostained with anti-MAP2.

### Lentivirus and RNA interference

The target sequence of shRNA against rat PLD1 was CTGGAAGATTACTTGACAA. The double strand DNA including the target sequence and shRNA loop were synthesized by annealing the two oligos: forward, 5′ CCGGCTGGAAGATTACTTGACAATTCAAGAGATTGTCAAGTAATCTTCCAGTTTTTTG 3′; reversed, 5′ AATTCAAAAAACTGGAAGATTACTTGACAATCTCTTGAATTGTCAAGTAATCTTCCAG 3′. Then the double strand DNA was subcloned into shRNA expressing vector pLKD.CMV.GFP.U6.shRNA. The packaging of lentivirus was done in the Obio Technology (Shanghai) Corp., Ltd. Briefly, the shRNA expressing vector was cotransfected with psPAX2 and pMD2.G constructs into HEK293t cells. After 2 days of transfection, the medium was collected and centrifuged by 100,000 g for 2 hours. Then the supernatant was discarded and the pellet was dissolved by opti-MEM (Invitrogen). The titer of the lentivirus reached 5 × 10^8^ IU/ml. The concentrated lentivirus was aliquot and stored in −80 °C.

### Western blot analysis

Astrocyte or neuronal cultures were washed twice with ice-cold PBS and incubated for 20 min in ice-cold lysis buffer (50 mM Tris-HCl, pH7.4, 150 mM NaCl, 1.5 mM MgCl_2_, 10% glycerol, 1% Triton X-100, 5 mM EGTA, 1 μg/ml leupeptin, 1 mM PMSF, 1 mM Na_3_VO_4_, 10 mM NaF, and proteinase inhibitor mixture). The lysates were centrifuged at 12,000 g for 5 min to yield the total protein extract in the supernatant. The concentration of protein was measured with a BCA assay kit (Pierce). Equal amounts of samples (50 μg) were denatured and subjected to 10% SDS-PAGE. After separation, proteins were transferred to nitrocellulose membranes (Bio-Rad). The membranes were blocked with 5% nonfat milk in TBST for 1 h at room temperature and incubated with primary antibody overnight at 4 °C. After washing with TBST for 3 times (5 min for each), the membranes were incubated with horseradish peroxidase (HRP)-conjugated secondary antibody for 1h at room temperature. After the membrane was washed with TBST for 4 times (5 min for each), it was developed with ECL solutions (ThermoFisher Scientific). The immunoreactive bands were scanned and analyzed quantitatively by densitometry with Quantity One (Bio-Rad).

### Measurement of phospholipids in astrocyte cultured medium

DIV 15 astrocyte culture medium was collected and centrifuged (6000 rpm × 5 min) to remove cell debris. Following which, fixed volume of supernatant was transferred to 10-ml glass vials. To each sample, 1 ml of ice-cold chloroform and 250 μl of 1 M HCl were added and vortexed for 5 min at room temperature. The samples were then centrifuged at 4000 rpm at 4 degree for 5 min. Lower organic phase was extracted using glass micropipettes and transferred to fresh tube. The extraction was repeated once with another 1 ml of ice-cold chloroform. The lipid extracts were pooled and dried in SpeedVac under OH mode. Samples were stored at −80 degree until further analysis of normal phase LC/MS.

### Normal phase LC/MS

Polar lipids were analyzed using an Agilent 1260 HPLC system coupled with a triple quadrupole/ion trap mass spectrometer (5500Qtrap; SCIEX) as described previously^39^. Separation of individual lipid classes of polar lipids by normal phase (NP)-HPLC was carried out using a Phenomenex Luna 3μ-silica column (internal diameter 150 × 2.0 mm) with the following conditions: mobile phase A (chloroform: methanol: ammonium hydroxide, 89.5:10:0.5) and mobile phase B (chloroform: methanol: ammonium hydroxide: water, 55: 39: 0.5: 5.5). MRM transitions were set up for comparative analysis of various polar lipids. Individual lipid species were quantified by referencing to spiked internal standards. PC-14:0/14:0, PE-14:0/14:0, PS-14:0/14:0, PA-17:0/17:0, PG-14:0/14:0, LPA-17:0 and LPC-17:0 were obtained from Avanti Polar Lipids (Alabaster, AL) and LIPID MAPS.

### Immunostaining

Hippocampal neurons on coverslips were washed with PBS and fixed with 4% paraformaldehyde for 20 min. Neurons were then washed with PBS for three times and permeabilized with 0.3% Triton X-100 for 30 min. After blocking with 10% goat serum, cells were incubated with primary antibodies overnight at 4 °C followed by Alexa Fluor-conjugated secondary antibodies (Molecular Probes) for 1 h at room temperature. Finally, the cells were mounted on slides, and the stained sections were examined with a Leica fluorescent microscope.

### Analysis of neuronal morphology

The morphologies of dendrites were indicated by the immunostaining of MAP2. We distinguish between pyramidal and inhibitory neurons form morphological appearance. GABAergic interneurons had more fusiform or polygonal shaped soma while pyramidal neurons had the characteristic triangular-shaped soma^40^. Pyramidal neurons were randomly chosen and their dendrites were traced under the Leica fluorescent microscope. The investigators were blind to the inhibitor treatment and shRNA expression. The analysis of dendritic branching was performed as previously described^14^. Briefly, all dendrites longer than 10 μm originating from the cell soma were defined as primary dendrites. All protrusions originating from the primary dendrites were defined as secondary dendrites. All terminal branches of dendrites with lengths more than 10 μm were counted as dendritic tips^41^. For Sholl analysis, concentric circles with 15 μm differences in diameter were drawn around the cell body, and the number of dendrites crossing each circle was manually counted^20^. All experiments were repeatedly performed at least by 3 times.

### Statistical analysis

All data are represented as mean ± SEM. Comparisons between two groups were made using t tests. Comparisons among three or more groups were made using one-way ANOVA analysis followed by Tukey tests. The statistics of the data from Sholl analysis were performed by two-way ANOVA. Data marked with asterisks are significantly different from the control as follows: ***p < 0.001, **p < 0.01, and *p < 0.05.

## Additional Information

**How to cite this article**: Zhu, Y.-B. *et al*. Astrocyte-derived phosphatidic acid promotes dendritic branching. *Sci. Rep.* **6**, 21096; doi: 10.1038/srep21096 (2016).

## Supplementary Material

Supplementary Information

## References

[b1] Clarke L. E. & Barres B. A. Emerging roles of astrocytes in neural circuit development. Nat Rev Neurosci 14, 311–321 (2013). 23595014 10.1038/nrn3484 PMC4431630

[b2] Jan Y. N. & Jan L. Y. Branching out: mechanisms of dendritic arborization. Nat Rev Neurosci 11, 316–328 (2010). 20404840 10.1038/nrn2836 PMC3079328

[b3] Noble M., Fok-Seang J. & Cohen J. Glia are a unique substrate for the *in vitro* growth of central nervous system neurons. J Neurosci 4, 1892–1903 (1984). 6737045 10.1523/JNEUROSCI.04-07-01892.1984 PMC6564884

[b4] Fallon J. R. Preferential outgrowth of central nervous system neurites on astrocytes and Schwann cells as compared with nonglial cells *in vitro*. J Cell Biol 100, 198–207 (1985). 3880751 10.1083/jcb.100.1.198 PMC2113471

[b5] Powell E. M., Meiners S., DiProspero N. A. & Geller H. M. Mechanisms of astrocyte-directed neurite guidance. Cell Tissue Res 290, 385–393 (1997). 9321702 10.1007/s004410050945

[b6] Tomaselli K. J., Neugebauer K. M., Bixby J. L., Lilien J. & Reichardt L. F. N-cadherin and integrins: two receptor systems that mediate neuronal process outgrowth on astrocyte surfaces. Neuron 1, 33–43 (1988). 2856086 10.1016/0896-6273(88)90207-3

[b7] Crone S. A. & Lee K. F. The bound leading the bound: target-derived receptors act as guidance cues. Neuron 36, 333–335 (2002). 12408835 10.1016/s0896-6273(02)01009-7

[b8] Araujo S. J. & Tear G. Axon guidance mechanisms and molecules: lessons from invertebrates. Nat Rev Neurosci 4, 910–922 (2003). 14595402 10.1038/nrn1243

[b9] Sung J. Y. . Differential activation of phospholipases by mitogenic EGF and neurogenic PDGF in immortalized hippocampal stem cell lines. J Neurochem 78, 1044–1053 (2001). 11553678 10.1046/j.1471-4159.2001.00491.x

[b10] Hayakawa K. . Increased expression of phospholipase D1 mRNA during cAMP- or NGF-induced differentiation in PC12 cells. Neurosci Lett 265, 127–130 (1999). 10327185 10.1016/s0304-3940(99)00228-1

[b11] Yoon M. S. . Role of phospholipase D1 in neurite outgrowth of neural stem cells. Biochem Biophys Res Commun 329, 804–811 (2005). 15752728 10.1016/j.bbrc.2005.02.087

[b12] Klein J. Functions and pathophysiological roles of phospholipase D in the brain. J Neurochem 94, 1473–1487 (2005). 16042758 10.1111/j.1471-4159.2005.03315.x

[b13] Burkhardt U. . Impaired brain development and reduced cognitive function in phospholipase D-deficient mice. Neurosci Lett 572, 48–52 (2014). 24813107 10.1016/j.neulet.2014.04.052

[b14] Zhu Y. B. . PLD1 Negatively Regulates Dendritic Branching. J Neurosci 32, 7960–7969 (2012). 22674271 10.1523/JNEUROSCI.5378-11.2012 PMC6620958

[b15] Ammar M. R. . The Coffin-Lowry syndrome-associated protein RSK2 regulates neurite outgrowth through phosphorylation of phospholipase D1 (PLD1) and synthesis of phosphatidic acid. J Neurosci 33, 19470–19479 (2013). 24336713 10.1523/JNEUROSCI.2283-13.2013 PMC6618760

[b16] Jin S. . Stability of phospholipase D in primary astrocytes. Biochem Biophys Res Commun 297, 545–551 (2002). 12270129 10.1016/s0006-291x(02)02231-3

[b17] Zeniou-Meyer M. . The Coffin-Lowry syndrome-associated protein RSK2 is implicated in calcium-regulated exocytosis through the regulation of PLD1. Proc Natl Acad Sci USA 105, 8434–8439 (2008). 18550821 10.1073/pnas.0710676105 PMC2448854

[b18] Siddhanta A. & Shields D. Secretory vesicle budding from the trans-Golgi network is mediated by phosphatidic acid levels. J Biol Chem 273, 17995–17998 (1998). 9660750 10.1074/jbc.273.29.17995

[b19] Choi S. Y. . A common lipid links Mfn-mediated mitochondrial fusion and SNARE-regulated exocytosis. Nat Cell Biol 8, 1255–1262 (2006). 17028579 10.1038/ncb1487

[b20] Sholl D. A. Dendritic organization in the neurons of the visual and motor cortices of the cat. J Anat 87, 387–406 (1953). 13117757 PMC1244622

[b21] Yin D. M., Huang Y. H., Zhu Y. B. & Wang Y. Both the establishment and maintenance of neuronal polarity require the activity of protein kinase D in the Golgi apparatus. J Neurosci 28, 8832–8843 (2008). 18753385 10.1523/JNEUROSCI.1291-08.2008 PMC6670825

[b22] Frohman M. A., Sung T. C. & Morris A. J. Mammalian phospholipase D structure and regulation. Biochim Biophys Acta 1439, 175–186 (1999). 10425394 10.1016/s1388-1981(99)00093-1

[b23] Yung Y. C., Stoddard N. C., Mirendil H. & Chun J. Lysophosphatidic Acid signaling in the nervous system. Neuron 85, 669–682 (2015). 25695267 10.1016/j.neuron.2015.01.009 PMC4400838

[b24] Kaech S. & Banker G. Culturing hippocampal neurons. Nat Protoc 1, 2406–2415 (2006). 17406484 10.1038/nprot.2006.356

[b25] Chen Y. G. . Phospholipase D stimulates release of nascent secretory vesicles from the trans-Golgi network. J Cell Biol 138, 495–504 (1997). 9245781 10.1083/jcb.138.3.495 PMC2141634

[b26] Siddhanta A., Backer J. M. & Shields D. Inhibition of phosphatidic acid synthesis alters the structure of the Golgi apparatus and inhibits secretion in endocrine cells. J Biol Chem 275, 12023–12031 (2000). 10766834 10.1074/jbc.275.16.12023

[b27] Guy A. T. . NEURONAL DEVELOPMENT. Glycerophospholipid regulation of modality-specific sensory axon guidance in the spinal cord. Science 349, 974–977 (2015). 26315437 10.1126/science.aab3516

[b28] Song H. J. & Poo M. M. Signal transduction underlying growth cone guidance by diffusible factors. Curr Opin Neurobiol 9, 355–363 (1999). 10395576 10.1016/s0959-4388(99)80052-x

[b29] Leem Y. H. . Repression of tau hyperphosphorylation by chronic endurance exercise in aged transgenic mouse model of tauopathies. J Neurosci Res 87, 2561–2570 (2009). 19360903 10.1002/jnr.22075

[b30] Wersinger C., Chen J. & Sidhu A. Bimodal induction of dopamine-mediated striatal neurotoxicity is mediated through both activation of D1 dopamine receptors and autoxidation. Mol Cell Neurosci 25, 124–137 (2004). 14962746 10.1016/j.mcn.2003.10.002

[b31] Guizzetti M., Zhang X., Goeke C. & Gavin D. P. Glia and neurodevelopment: focus on fetal alcohol spectrum disorders. Front Pediatr 2, 123 (2014). 25426477 10.3389/fped.2014.00123 PMC4227495

[b32] Burkhardt U., Beyer S. & Klein J. Role of phospholipases D1 and 2 in astroglial proliferation: effects of specific inhibitors and genetic deletion. Eur J Pharmacol (2015). 10.1016/j.ejphar.2015.05.004 25967349

[b33] Cockcroft S. & Phospholipase D. regulation by GTPases and protein kinase C and physiological relevance. Prog Lipid Res 35, 345–370 (1996). 9246355 10.1016/s0163-7827(96)00009-4

[b34] Zhang Y., Kanaho Y., Frohman M. A. & Tsirka S. E. Phospholipase D1-promoted release of tissue plasminogen activator facilitates neurite outgrowth. J Neurosci 25, 1797–1805 (2005). 15716416 10.1523/JNEUROSCI.4850-04.2005 PMC6725938

[b35] Humeau Y. . A role for phospholipase D1 in neurotransmitter release. Proc Natl Acad Sci USA 98, 15300–15305 (2001). 11752468 10.1073/pnas.261358698 PMC65024

[b36] Wang X., Devaiah S. P., Zhang W. & Welti R. Signaling functions of phosphatidic acid. Prog Lipid Res 45, 250–278 (2006). 16574237 10.1016/j.plipres.2006.01.005

[b37] Tan Z. J., Peng Y., Song H. L., Zheng J. J. & Yu X. N-cadherin-dependent neuron-neuron interaction is required for the maintenance of activity-induced dendrite growth. Proc Natl Acad Sci USA 107, 9873–9878 (2010). 20457910 10.1073/pnas.1003480107 PMC2906874

[b38] Yu A. C., Lee Y. L. & Eng L. F. Astrogliosis in culture: I. The model and the effect of antisense oligonucleotides on glial fibrillary acidic protein synthesis. J Neurosci Res 34, 295–303 (1993). 8455207 10.1002/jnr.490340306

[b39] Lam S. M. . Extensive characterization of human tear fluid collected using different techniques unravels the presence of novel lipid amphiphiles. J Lipid Res 55, 289–298 (2014). 24287120 10.1194/jlr.M044826 PMC3886667

[b40] Benson D. L., Watkins F. H., Steward O. & Banker G. Characterization of GABAergic neurons in hippocampal cell cultures. J Neurocytol 23, 279–295 (1994). 8089704 10.1007/BF01188497

[b41] Jaworski J., Spangler S., Seeburg D. P., Hoogenraad C. C. & Sheng M. Control of dendritic arborization by the phosphoinositide-3′-kinase-Akt-mammalian target of rapamycin pathway. J Neurosci 25, 11300–11312 (2005). 16339025 10.1523/JNEUROSCI.2270-05.2005 PMC6725892

