# Supplementary material for: Astrocyte-derived phosphatidic acid promotes dendritic branching
Source: Sci Rep. 2016 Feb 17;6:21096. doi: 10.1038/srep21096 (PMC4756377; doi:10.1038/srep21096)
Supplement: Supplementary Information [file srep21096-s1.pdf]

Astrocyte-derived phosphatidic acid promotes dendritic branching

Yan-Bing Zhu[1#], Weizhen Gao[2#], Yongbo Zhang[1#], Feng Jia[2],
Hai-Long Zhang[3], Ying-Zi Liu[3], Xue-Fang Sun[3], Yuhua Yin[1] and Dong-Min Yin[3*]

Figure S1

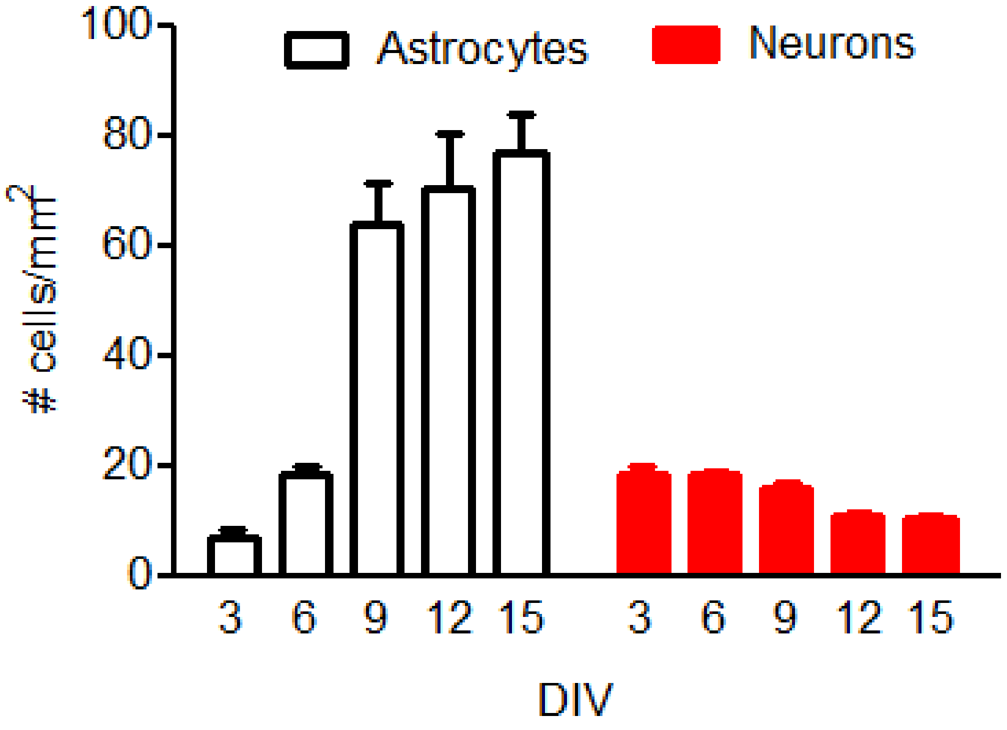
Astrocytes
Neurons
cells/mm^2
100
80
60
40
20
0
3
6
9
12
15
3
6
9
12
15
DIV

Figure S2

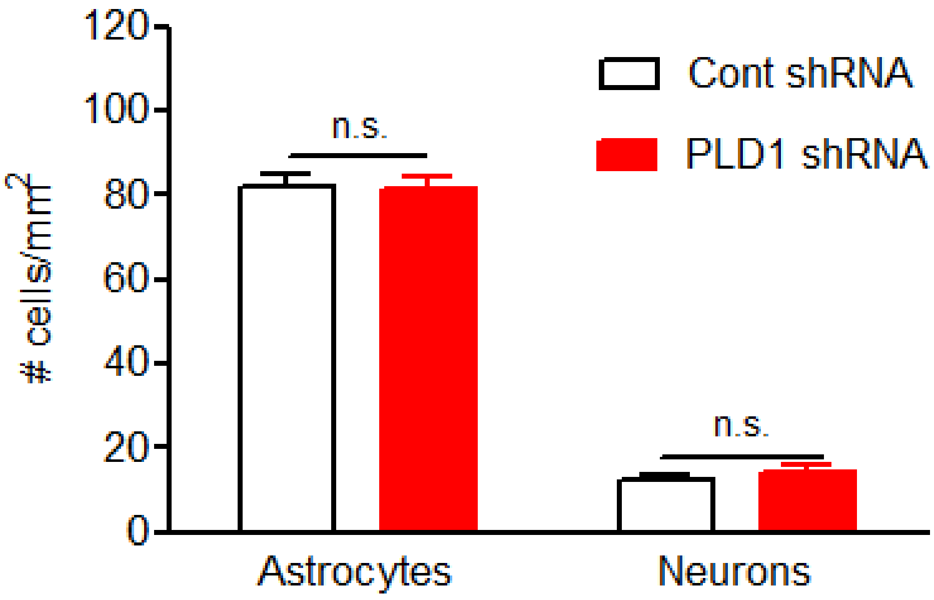

120
100
80
60
40
20
0
cells/mm2
n.s.
n.s.
Cont shRNA
PLD1 shRNA
Astrocytes
Neurons

Figure S3

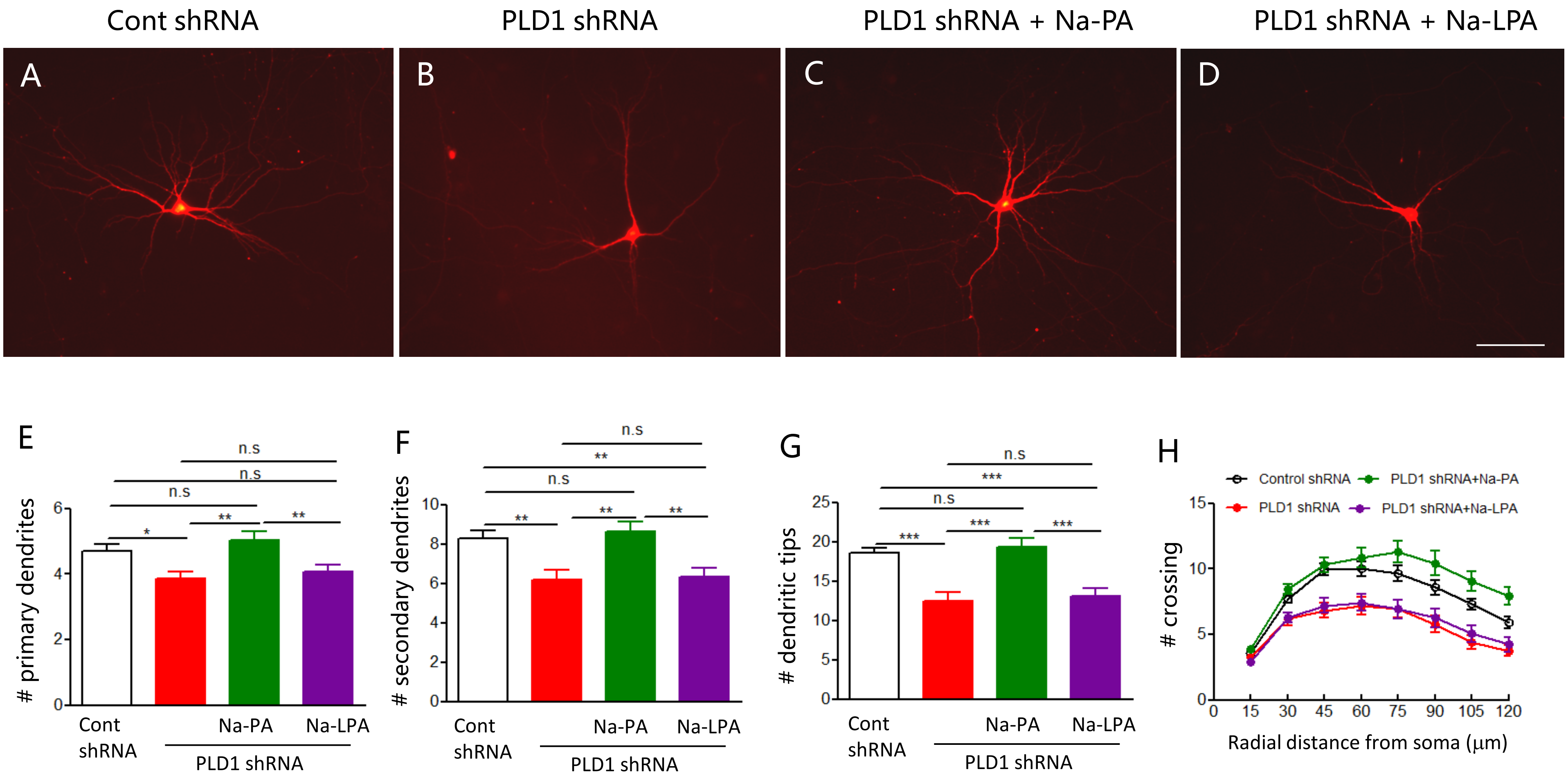

Cont shRNA
PLD1 shRNA
PLD1 shRNA + Na-PA
PLD1 shRNA + Na-LPA
A
B
C
D
E
primary dendrites
n.s
n.s
n.s
*
**
**
Cont shRNA
Na-PA
Na-LPA
PLD1 shRNA
F
secondary dendrites
n.s
**
n.s
**
**
**
Cont shRNA
Na-PA
Na-LPA
PLD1 shRNA
G
dendritic tips
n.s
***
n.s
***
***
***
Cont shRNA
Na-PA
Na-LPA
PLD1 shRNA
H
Control shRNA
PLD1 shRNA+Na-PA
PLD1 shRNA
PLD1 shRNA+Na-LPA
crossing
Radial distance from soma (μm)

Figure S4

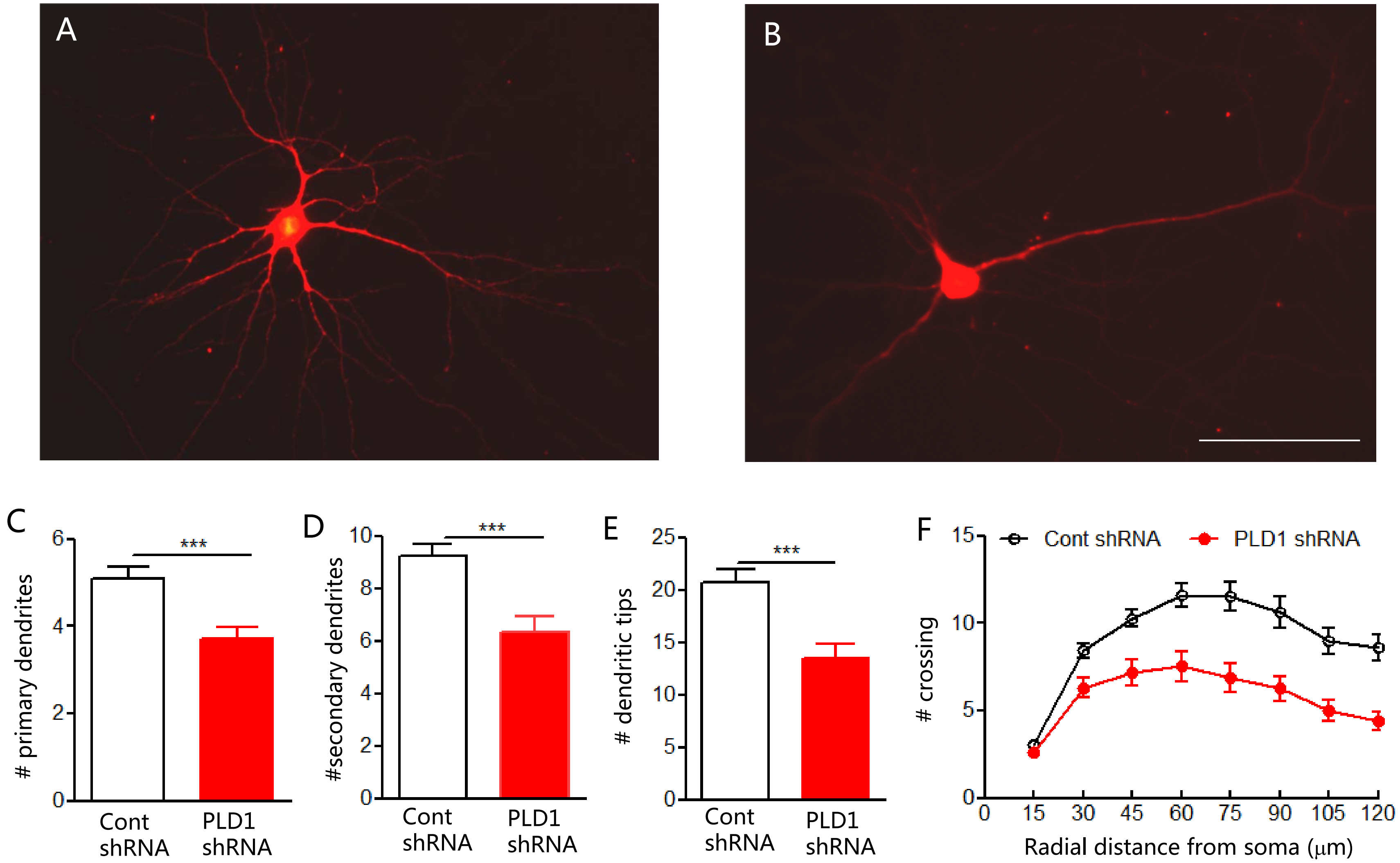

A
B
C
primary dendrites
6
4
2
0
***
Cont shRNA
PLD1 shRNA
D
#secondary dendrites
10
8
6
4
2
0
***
Cont shRNA
PLD1 shRNA
E
dendritic tips
25
20
15
10
5
0
***
Cont shRNA
PLD1 shRNA
F
Cont shRNA
PLD1 shRNA
crossing
15
10
5
0
0 15 30 45 60 75 90 105 120
Radial distance from soma (μm)

Figure S5

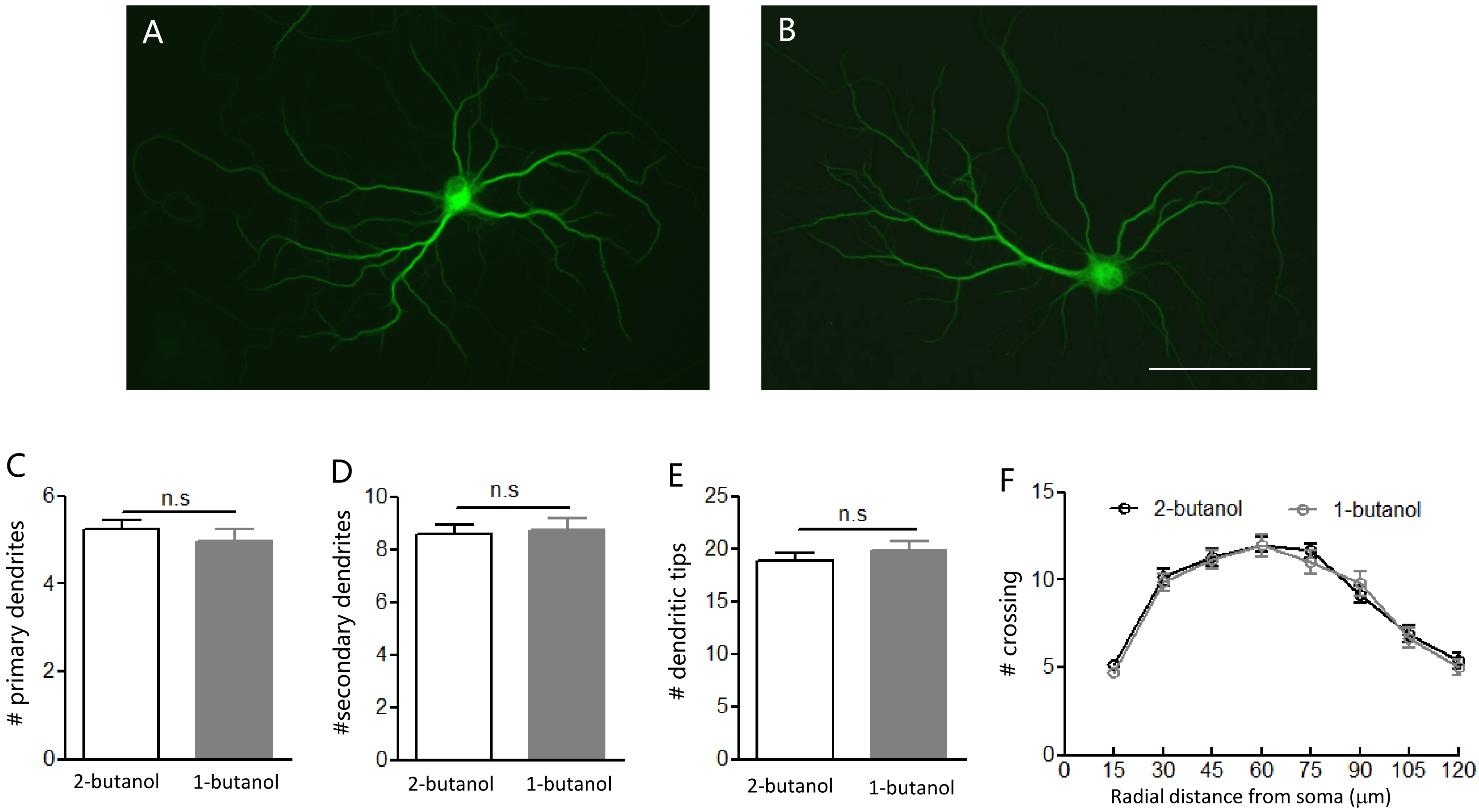

A
B
C
primary dendrites
n.s
2-butanol
1-butanol
D
#secondary dendrites
n.s
2-butanol
1-butanol
E
dendritic tips
n.s
2-butanol
1-butanol
F
crossing
2-butanol
1-butanol
Radial distance from soma (μm)

Figure legends

Figure S1 Number of astrocytes and neurons in neuron-glia mixed culture at the indicated different time points.

Figure S2 Number of astrocytes and neurons in DIV 15 neuron-glia mixed culture transduced by lentivirus expressing control and PLD1 shRNA.

Figure S3 Dendritic branching of neurons in neuron-glia mixed culture. **A-D,** dsRed of DIV 15 neurons in neuron-glia culture where only astrocytes were infected by lentivirus expressing control shRNA, PLD1 shRNA and PLD1 shRNA plus 1 μM Na-PA or Na-LPA in the culturing medium. Bar, 100 μm. **E-H,** Quantification of primary dendrites (E), secondary dendrites (F), dendritic tips (G) and Sholl analysis (H) for four groups of neurons in panel A to D, n = 53 neurons for control shRNA, n = 56 neurons for PLD1 shRNA, n = 64 neurons for PLD1 shRNA plus Na-PA, n = 66 neurons for PLD1 shRNA plus Na-LPA from 3 independent experiments, E-G, * $p < 0.05$, ** $p < 0.01$, *** $p < 0.001$, H, $F(3, 1320) = 58.72$, $p < 0.001$.

Figure S4 Dendritic branching of neurons in neuron-glia mixed culture with normal density. **A-B,** dsRed in DIV 15 neurons in neuron-glia culture where only astrocytes were infected by lentivirus expressing control shRNA and PLD1 shRNA. Bar, 100 μm. **C-F,** Quantification of primary dendrites (C), secondary dendrites (D), dendritic tips (E) and Sholl analysis (F) for two groups of neurons in panel A to B, n = 58 neurons for control shRNA, n = 59 neurons for PLD1 shRNA from 3 independent experiments, C-E, *** $p < 0.001$, F, $F(1, 560) = 95.98$, $p < 0.001$.

Figure S5 Dendritic branching of neurons in pure neuronal culture under treatment with vehicle and PLD1 inhibitor. **A,** MAP2 staining of DIV 15 neurons treated with 0.5% 2-butanol. **B,** MAP2 staining of DIV 15 neurons treated with 0.5% 1-butanol. Bar, 100 μm. **C-F,** Quantification of primary dendrites (C), secondary dendrites (D), dendritic tips (E) and Sholl analysis (F) for two groups of neurons in panel A and B, n = 58 for neurons treated with 2-butanol , n = 61 for neurons treated with 1-butanol from 3 independent experiments.
